# Impact of COVID-19 School Closures on White Matter Plasticity in the Reading Network

**DOI:** 10.1162/nol_a_00158

**Published:** 2025-01-10

**Authors:** Lauren Blockmans, Fumiko Hoeft, Jan Wouters, Pol Ghesquière, Maaike Vandermosten

**Affiliations:** Research Group ExpORL, Leuven Brain Institute, Department of Neurosciences, KU Leuven, Leuven, Belgium; Department of Psychological Sciences, University of Connecticut, Storrs, CT, USA; Parenting and Special Education Research Unit, Faculty of Psychology and Educational Sciences, KU Leuven, Leuven, Belgium

**Keywords:** COVID-19 school closures, diffusion MRI, neural reading network, phonological awareness, pre-reading

## Abstract

During the COVID-19 pandemic, children worldwide experienced school closures. Several studies have detected a negative impact on reading-related skills in children who experienced these closures during the early stages of reading instruction, but the impact on the reading network in the brain has not been investigated. In the current longitudinal study in a sample of 162 Dutch-speaking children, we found a short-term effect in the growth of phonological awareness in children with COVID-19 school closures compared to children without school closures, but no long-term effects one year later. Similarly, we did not find a long-term effect on the longitudinal development of white matter connectivity in tracts implicated during early reading development. Together, these findings indicate that one year after school closures no effects on the development of phonological awareness and white matter are found, yet it remains an open question whether short-term effects on the reading network could have been present and/or whether other networks (e.g., psychosocial related networks) are potentially more affected.

## INTRODUCTION

Acquiring reading skills is essential for later academic achievement and socioemotional well-being (Haft et al., 2016; Livingston et al., 2018). Unlike oral language skills, which are acquired spontaneously, written language skills require formal instruction, which is usually initiated in the first grade of primary school. Recently, in response to the COVID-19 pandemic, schools worldwide were closed in an effort to slow down the spread of the new coronavirus (SARS-CoV-2), resulting in more than one billion children having to stay home (Conto et al., 2021; UNESCO, 2022) for 20 weeks on average (UNICEF, 2022). In this study, we investigated the effect of COVID-19 school closures on the development of reading-related skills and structural brain connectivity within the reading network in children who experienced school closures during a pivotal time of reading acquisition (first grade) and of whom a majority was at risk for developmental dyslexia.

### Impact of School Closures on Reading Development

Long periods of interrupted schooling, for example, in the context of natural events (Bennett, 2022; Goodman, 2014; Marcotte & Hemelt, 2008; Miller & Hui, 2022; Sapkota & Neupane, 2021; Thamtanajit, 2020), economic hardship with associated absenteeism (Dolean et al., 2019; Paschall et al., 2018; Ready, 2010), and childhood abuse (Snow, 2021), have repeatedly been shown to negatively affect academic achievement, including, but not limited to, reading abilities. Similarly, the impact of schooling disruption has been demonstrated in studies investigating summer loss that reported larger variation in (pre-)reading skills during the summer holidays than during the school year (Downey et al., 2004; Luyten et al., 2023; Quinn et al., 2016; von Hippel et al., 2018). Several studies also reported a loss of reading skills after two months of summer holidays across primary school grades (Atteberry & McEachin, 2021; Cooper et al., 1996). Summer learning loss is believed to increase reading skill gaps especially between children with different socioeconomic backgrounds. Indeed, Alexander et al. (2007) and von Hippel et al. (2018) found a more detrimental impact in children with lower socioeconomic status (SES) compared to children with higher SES. Importantly, some studies do not find clear evidence for summer learning loss of reading skills (Shinwell & Defeyter, 2017). Across studies, reading has been measured using different testing methods (fixed test across time or adaptive test) and different scaling methods (e.g., nonscaled accuracy scores or scaled scores based on Item Response Theory). It has been argued that such measurement differences could explain the diverging results reported across studies (von Hippel & Hamrock, 2019; Workman et al., 2023). Nevertheless, available evidence suggests a possible negative impact of summer holidays on reading development.

In light of these negative effects of interrupted schooling, a similar negative impact of school closures during the COVID-19 pandemic could be expected, especially in the context of reading acquisition, as it requires formal reading instruction. At the onset of the pandemic, estimations based on absenteeism and summer loss studies already predicted large losses in reading skills (Bao et al., 2020; Kuhfeld, Soland, et al., 2020). As the pandemic progressed, empirical data demonstrated reading losses of 0.08 standard deviations (*SD*) in The Netherlands (Engzell et al., 2021), 0.19 *SD* in Belgium (Maldonado & De Witte, 2022), 0.07 *SD* in Germany (Schult et al., 2022) and up to 0.17 *SD* in the US (Kuhfeld et al., 2023). In Finland, increasing effects of school closures on reading were found with advancing grades, from effect size *d* = 0.26 in first grade, to *d* = 0.42 in second grade and *d* = 0.60 in fourth grade (Lerkkanen et al., 2023). This stands in contrast to the larger losses in academic achievement, including reading comprehension, reported in younger children (9- to 12-year-olds) compared to older children (13- to 15-year-olds; Tomasik et al., 2021). While some studies did not find any evidence for reading loss (Depping et al., 2021; Förster et al., 2023; Gore et al., 2021; Kuhfeld, Tarasawa, et al., 2020), systematic reviews concluded that the majority of evidence points toward substantial losses (see, e.g., Donnelly & Patrinos, 2022, and Hammerstein et al., 2021). More recently, a median effect size of *d* = 0.09 (equivalent to a loss of 0.09 *SD*) was found for reading in a comprehensive meta-analysis including 42 studies from all over the world (Betthäuser et al., 2023).

A crucial factor during reading development is the awareness of speech sounds in spoken words (phonological awareness, PA), which has been related to typical and atypical reading development (Melby-Lervåg et al., 2012; Quinn et al., 2015). Very few studies examined the impact of school closures on PA specifically, with only a couple of studies reporting losses in the context of summer holidays (Skibbe et al., 2012; Tiruchittampalam et al., 2018). In the context of COVID-19 school closures, the findings have been inconsistent. While Sun et al. (2023) found no significant effect, Coskun and Kara (2022) reported a negative impact. Even though formal reading instruction in primary school might not explicitly include PA training, the bidirectionality between PA and reading (Clayton et al., 2020; Peterson et al., 2018) could explain why interrupted schooling also affects PA (Peng & Kievit, 2020). In sum, evidence concerning interrupted schooling effects on PA remains limited and requires further investigation.

### Brain Development During Reading Acquisition

Children acquire reading skills during the first few years of primary school, a time when gray and white matter across the whole brain is still developing considerably (Gilmore et al., 2018; Groeschel et al., 2010; Lebel & Beaulieu, 2011). In the past decades, many neuroimaging studies identified specific structural and functional brain changes that are associated with the acquisition of reading skills (Chyl et al., 2021). These changes include the left hemispheric reading network, consisting of three main gray matter regions, that is, inferior-frontal, temporoparietal and occipitotemporal regions (Martin et al., 2015), and the long-range white matter connections between those regions, such as the arcuate fasciculus (AF) and the inferior fronto-occipital fasciculus (IFOF) (Kearns et al., 2019). Moreover, characteristics of the reading network are known to differentiate between individuals with typical reading skills and individuals with severe reading difficulties (dyslexia), even before the onset of reading acquisition (Chyl et al., 2021; D’Mello & Gabrieli, 2018; Norton et al., 2015; Ozernov-Palchik & Gaab, 2016). It has been suggested that some differences are driven by the presence of a family risk for dyslexia, regardless of the eventual reading outcomes. Indeed, a few neuroimaging studies have described a family risk dependent pattern for structural features (Beelen et al., 2019; Hakvoort et al., 2015; Plakas et al., 2013; Vanderauwera et al., 2018; van Zuijen et al., 2012) and for functional activation patterns (Łuniewska et al., 2019; Vandermosten et al., 2020) of bilateral temporal regions such as the temporal gyri and the planum temporale, which have been considered important for the processing of auditory stimuli and speech sounds.

Across development, gray matter changes within the reading network were reported to be more prominent during early reading stages (kindergarten to second grade) compared to advanced reading stages (second to fifth grade), reflecting the brain dynamics during these early stages (Phan et al., 2021). Moreover, the relationship between actual reading skills and gray matter properties in the reading network seems to be dynamic across development, with brain-driven changes in reading skills in the pre-reading stage (kindergarten; Beelen et al., 2019) and behavior-driven brain changes in later stages (second to fifth grade; Beelen et al., 2022). In a similar vein, the direction of associations between reading skills and white matter organization seems to vary across reading development (Meisler & Gabrieli, 2022; Yeatman, Dougherty, Ben-Shachar, & Wandell, 2012). Possibly, such dynamics during development are associated with changes in the environment (Wandell & Yeatman, 2013). Studies have found white matter changes in the case of increased input within the educational environment as provided by experimental reading interventions (Economou et al., 2023; Huber et al., 2021), hence brain changes could also be expected with decreased input from the environment, for example, due to COVID-19 lockdowns. Although not specific to reading development, two studies have described an influence of COVID-19 lockdowns on adolescents’ brain maturation of gray matter in the bilateral hippocampus and amygdala (Gotlib et al., 2022) and in the hippocampus and prefrontal cortex (but not in the amygdala; van Drunen et al., 2023). Hence, school closures during a pivotal time of acquiring reading skills might influence brain development in the reading network. However, evidence in younger children specific to the brain network involved during reading acquisition is currently lacking.

### The Current Study

In the current study, we investigated the effect of COVID-19 school closures on the development of PA and white matter tracts implicated in reading. To this end, we compared two cohorts of children that followed an identical longitudinal research protocol (from kindergarten until third grade), with the crucial difference that one cohort experienced school closures during first grade whereas the other cohort did not. As a result, the comparison of the two cohorts provided a unique opportunity to investigate the impact of school closures. PA was assessed in-person at the beginning of first grade (before school closures), and at the beginning of second grade (directly after school closures), and at the beginning of third grade (more than one year after school closures). In line with Coskun and Kara (2022) and Peng and Kievit (2020), we expected a negative impact of the school closures on the growth of PA. Although the evidence has been conflicting (Lerkkanen et al., 2023; Tomasik et al., 2021), we also expected larger negative effects on the assessment of PA directly after school closures (second grade) compared to the assessment that took place one year later (third grade). Structural magnetic resonance imaging (MRI) was acquired once at the end of kindergarten (before school closures) and once at the end of second grade (one year after school closures). We expected a general increase in white matter organization of the bilateral AF and IFOF over time (Lebel & Beaulieu, 2011). However, we also expected that the COVID-19 pandemic and associated school closures affected the reading network in children who experienced school closures during a pivotal time of acquiring reading skills (first grade). More specifically, we expected that children of this cohort would show less of the typical maturational increase in white matter organization of the bilateral AF and IFOF. The expected effect on the brain is rooted in at least two studies showing a COVID effect on the adolescent brain (Gotlib et al., 2022; van Drunen et al., 2023), yet note that although these studies found *accelerated* maturation (potentially related to increased stress levels during COVID), we expect to find *delayed* maturation (related to decreased training of academic skills during COVID).

## MATERIALS AND METHODS

### Participants

This longitudinal study is part of a DYSCO-project (DYSlexia Collaboration, KU, Leuven) that followed up two cohorts of children throughout their early reading development. Both cohorts were recruited at the start of the third and final year of kindergarten in Flanders (Belgium). The first cohort was recruited in 2011 and included 87 children, half of whom had an increased familial risk for dyslexia, defined as having at least one first-degree relative with a dyslexia diagnosis. For the second cohort, we recruited 76 children from a large-scale screening in 2018 (*n* = 1,225; Verwimp et al., 2020). Most of the children in the second cohort did not have a family risk but were selected to have an increased cognitive risk, defined as scoring below the 30th percentile on at least two out of three pre-reading tasks (PA, rapid automatized naming, and letter knowledge). Retrospectively, we also assessed cognitive risk in the 2011 cohort. These recruitment efforts resulted in the total sample (*n* = 163), consisting of 27 children with only a family risk (25 in the first and 2 in the second cohort), 64 children with only a cognitive risk (10 in the first and 54 in the second cohort), 36 children with a combined family and cognitive risk (20 in the first and 16 in the second cohort), and 36 children without any risk (32 in the first and 4 in the second cohort). In both cohorts, participants were monolingual Dutch-speakers and did not have a risk for ADHD nor any history of hearing loss, vision deficits, or brain damage. Data from one participant were excluded from all analyses due to an incidental brain finding, resulting in a total study sample of 162 participants.

All participants followed an identical study design, with the same measures administered at the same time points throughout development. More specifically, PA was assessed in schools at the start of first, second, and third grade, and structural brain images were acquired once at the end of the third year of kindergarten and once at the end of second grade. In Flanders, reading instruction is not yet formally initiated in kindergarten in line with governmental regulations (https://onderwijsdoelen.be/), hence kindergarten is considered the pre-reading stage. For the 2011 cohort, all these measures were collected prior to the COVID-19 pandemic. For the 2018 cohort, PA at the start of first grade and pre-reading brain imaging also occurred before the COVID-19 pandemic. However, children in this cohort experienced school closures during first grade, since the National Security Council in Belgium ordered all primary and secondary schools to close starting from March 16, 2020. Reopening was allowed partially from May 18, 2020, and full time from June 8, 2020. During the school closures, schools were responsible for offering remote teaching activities and/or online exercises (Maldonado & De Witte, 2022). In the current study, PA at the start of second and third grade, and brain imaging data at the end of second grade were collected after the COVID-19 school closures for the 2018 cohort. This difference between the cohorts allowed us to investigate the influence of school closures on PA and white matter development.

### PA

PA was assessed using a phoneme deletion task at the start of first, second, and third grade (Boets et al., 2010). In this task, monosyllabic nonexisting words (CCVC or CVCC) were presented orally. The participants were instructed to delete a phoneme from the presented nonwords. The task was divided in two sections, including two practice items prior to each section. The first section consisted of 10 test items of which the phoneme deletion resulted in an existing word. The second section consisted of 18 test items which remained nonexisting words after deletion of the phoneme. The maximum accuracy score on this task was 28.

### Brain Imaging Acquisition

Brain imaging data were collected at the end of kindergarten (pre-reading stage) and at the end of second grade using MRI. Prior to the MRI appointment, we provided the children with an informative video to familiarize them with the procedure of an MRI scan. During the actual MRI appointment at the University Hospital of Leuven, we implemented a child-friendly protocol to prepare the child. This protocol included in-person minigames to provide them with guidelines regarding communication during the scan and avoiding head motion (Theys et al., 2014). The MRI images were acquired in a 3T Philips Achieva scanner (Philips, Best, The Netherlands) equipped with a 32-channel head coil. Note that the same MRI sequences were acquired in the same type of scanner for both cohorts, but the scanner was replaced after both MRI data collection waves of the 2011 cohort, hence the scanner itself was different between the cohorts. For both cohorts, we acquired a diffusion-weighted sequence with the following parameters: sagittal diffusion imaging slices, 7,600 ms repetition time, 65 ms echo time, 90° flip angle, 2.5 × 2.5 × 2.5 mm voxel size, 60 noncollinear directions, 1,300 s/mm^2^ b-value, 6 non-diffusion weighted images and 10 min 32 s acquisition time. In addition, we acquired a T1-weighted sequence with following parameters: 182 contiguous coronal slices, 9.7 ms repetition time, 4.61 ms echo time, 8° flip angle, 0.98 × 0.98 × 1.20 mm voxel size and 6 min 22 s acquisition time.

The diffusion-weighted images were preprocessed in FSL (Version 6.0; Jenkinson et al., 2012), including a motion and Eddy currents correction. From this, we extracted a motion parameter for each participant at each time point by calculating the root mean square of the translational and rotational displacements in three directions (*x*, *y*, and *z* axes) for each volume of the scan, relative to the first volume. Scans with excessive motion, that is, scans with a motion parameter higher than half the voxel size (2.5/2 = 1.25 mm), were excluded from further analyses. Excessive head motion was present in one scan at the end of kindergarten and one scan at the end of second grade. After applying motion exclusion, MRI scans were available for 122 participants at the end of kindergarten and for 118 participants at the end of second grade. Of these children, 106 had scans at both time points.

T1-weighted images were aligned to the anterior commissure-posterior commissure line, which were then used to align the diffusion images using mrVista (Vista Lab, 2015) in Matlab R2019b (MathWorks, 2018). This served as input for the Automated Fiber Quantification pipeline (AFQ; Yeatman, Dougherty, Myall, et al., 2012), implemented in Matlab R2019b. In short, AFQ identified fiber groups in the whole brain applying a deterministic algorithm, which were assigned to white matter tracts defined by regions of interest. Then, the fiber groups were refined based on a probability map in native space and cleaned iteratively based on the length of the tract and the distance from the core of the tract. Although AFQ also provides the option to clip the tracts, we did not include this step, in line with other studies of our research group (Economou et al., 2022; Vandecruys et al., 2024; Van Der Auwera et al., 2021), since we wanted to take into account possible differences in the cortical endpoints of the tracts. Then, AFQ resampled each tract into 100 equidistant nodes and quantified diffusion indices at each of the 100 nodes as a weighted average over all of the fibers of the tract (Tract Profile). This weighting depended on the distance of the fibers from the core of the tract. More details of the AFQ-procedure can be found in Yeatman, Dougherty, Myall, et al. (2012). From AFQ, we extracted fractional anisotropy (FA) of two of the major white matter tracts implicated in reading development, that is, bilateral AF and IFOF. Although these two tracts were the main focus of our study, for completeness we also provide results of inferior longitudinal fasciculus (ILF) in Supporting Information (see Supplementary Material 3, available at https://doi.org/10.1162/nol_a_00158), given that ILF has also been linked to reading-related skills (Yeatman, Dougherty, Ben-Shachar, & Wandell, 2012; but see Saygin et al., 2016). In line with previous developmental studies on reading (Economou et al., 2022; Langer et al., 2017; Van Der Auwera et al., 2021; Wang et al., 2017), AFQ was not able to reconstruct the selected tracts (AF and IFOF) for all children, with especially a higher proportion of failed reconstructions in right AF (kindergarten: left AF: 15%, right AF: 49%, left IFOF: 20%, right IFOF: 17%; Grade 2: left AF: 20%, right AF: 39%, left IFOF: 8%, right IFOF: 10%). In addition, AFQ could not quantify FA at the first and last node of these tracts in some subjects, hence only nodes 2–99 were included in our analyses. For each of the tracts, the average FA-value over nodes 2–99 was used in the main statistical analyses. Given the relatively recent development of node-based (Tract Profile) analyses and theoretical constraints regarding the interpretation of localized findings (Ramus et al., 2018), we have chosen to report methodological details and results of the Tract Profile analyses only in the Supporting Information (see Supplementary Material 2).

### Statistical Analyses

All statistical analyses were performed in R (Version 4.1.2; R Core Team, 2021). The analyses script and anonymized data sheet are available at https://osf.io/cekp6/. Note that the conditions of our ethics approval do not permit public archiving raw MRI data, since consent had been obtained only for participation in the study, and not for sharing data with third parties. In order to obtain the data on request, a data sharing agreement must be signed and participants must be contacted again to provide consent for data sharing. Alternatively, requestors have to be affiliated as KU Leuven employee, but data have to stay at the KU Leuven server by all means.

First, we checked group differences between the two cohorts. More specifically, we checked whether the distribution of sex, age, handedness, head motion, family risk for reading difficulties, and parental education differed between the cohorts. In the main analyses for white matter, head motion was included as covariate given its known influence on FA (Baum et al., 2018; Yendiki et al., 2014). In addition, all analyses were re-run with other variables that tended to differ significantly (*p* < 0.10) between the cohorts included simultaneously as covariates to check if the results were impacted by these possible confounders.

For the main analyses, we first investigated whether the change in PA from first to second to third grade differed between the two cohorts. For the 2018 cohort the first grade time point is before the school closures, the second grade time point closely after the school closures and the third grade time point more than one year after the school closures. We fitted a linear mixed effects model with PA as dependent variable, time point (three levels: first grade, second grade, and third grade), cohort (two levels: 2011 cohort and 2018 cohort) and cohort-by-time interaction as predictors. This model was fitted using the lmer function of the lmerTest package (Kuznetsova et al., 2017). Concerning white matter, we first harmonized the FA-values of each of the four tracts (i.e., left and right AF and IFOF) using NeuroComBat (Fortin et al., 2017), which enables control for potential differences in the scanner between cohorts (i.e., the 2011 cohort was scanned on the same type of scanner and with the same acquisition sequence as the 2018 cohort, but the scanner itself was different). Results without harmonization are provided in Supplementary Material 1. To investigate the school closure effect on white matter development, we fitted linear mixed effects models to the tract average (harmonized) FA of each of the four tracts under study with random intercepts for subject. Time point (two levels: end of kindergarten and end of second grade), cohort (two levels: 2011 cohort and 2018 cohort), and their interaction were included as fixed effects. Head motion was included as covariate. In Supplementary Material 2, node-based (Tract Profile) analyses can be found. *F* statistics and *p* values were estimated using type III sum of squares and the Satterthwaite method. Standardized estimates were reported and interpreted as small, typical, or large effects whenever the estimates were larger than 0.10, 0.20, or 0.30, respectively (Gignac & Szodorai, 2016). Given that AFQ was not able to reconstruct the selected tracts for all children (with failed reconstructions ranging from 8% to 49% across tracts), we performed additional analyses using imputation techniques (see Supplementary Material 6) to ensure that the obtained pattern of results was robust against these missing data.

Finally, normality of the model residuals was checked using the Shapiro-Wilk test. In case of non-normal residuals, we applied robust linear mixed models (robustlmm package; Koller, 2016) using parametric bootstrapping (afex package; Singmann et al., 2023).

## RESULTS

In Table 1, general characteristics of the participants are shown, that is, sex, handedness, age, head motion during brain imaging, family risk, and parental education. For each of these participant characteristics, descriptive statistics of the total study sample as well as a comparison between the two cohorts is shown. Motion is included as covariate to the main analyses of white matter. Table 1 further showed significant differences between the two cohorts for sex, age at the end of kindergarten, and parental education as well as a trend for family risk. The main analyses were re-run including sex, family risk, and parental education simultaneously as additional covariates in the PA analyses and including sex, family risk, parental education, and age at the end of kindergarten simultaneously as additional covariates in the white matter analyses to check if the pattern of results changed. Results are described at the end of each paragraph below.

**Table 1.** Participant characteristics.

Variable	Cohort			*p* value^e^
	Overall (*n* = 162)^a^	2011 (*n* = 87)^a^	2018 (*n* = 75)^a^	
Sex (female/male)	80/82	36/51	44/31	0.028
Handedness (left/right)^b^	16/107	9/59	7/48	0.934
Age (months) - MRI end of kindergarten	73 (66–81)	74 (68–81)	72 (66–78)	0.002
Age (months) - PA begin of 1st grade	74 (68–81)	74 (68–81)	74 (69–79)	0.619
Age (months) - PA begin of 2nd grade	86 (80–92)	86 (80–92)	86 (80–91)	0.990
Age (months) - MRI end of 2nd grade	96 (90–102)	95 (90–101)	96 (90–102)	0.146
Age (months) - PA begin of 3rd grade	98 (92–105)	98 (93–105)	98 (92–104)	0.451
Motion at kindergarten MRI (mm)	0.53 (0.22–1.18)	0.52 (0.25–1.03)	0.54 (0.22–1.18)	0.626
Motion at 2nd grade MRI (mm)	0.49 (0.18–1.04)	0.47 (0.21–0.99)	0.51 (0.18–1.04)	0.080
Family risk^c^	0.32 (0.09–0.57)	0.33 (0.13–0.56)	0.30 (0.09–0.57)	0.095
Parental education^d^	4.03 (2.00–6.00)	3.79 (2.00–6.00)	4.24 (2.00–6.00)	0.039

*Note*. MRI = magnetic resonance imaging, PA = Phonetic awareness.

^a^ Mean (range) or occurrence (*n*).

^b^ Handedness was evaluated using the parents’ report of the Edinburgh Handedness Inventory.

^c^ Family risk for reading difficulties was quantified with the Dutch translation (Blockmans et al., 2023) of the parental Adult Reading History Questionnaire (ARHQ; Lefly & Pennington, 2000).

^d^ Parental education was quantified as the sum of the maternal and paternal highest level of education.

^e^ Group differences were assessed using a Pearson’s Chi-squared test for sex, a Fisher’s exact test for handedness and a Wilcoxon rank sum test for age, motion, family risk, and parental education.

**Figure 1.**
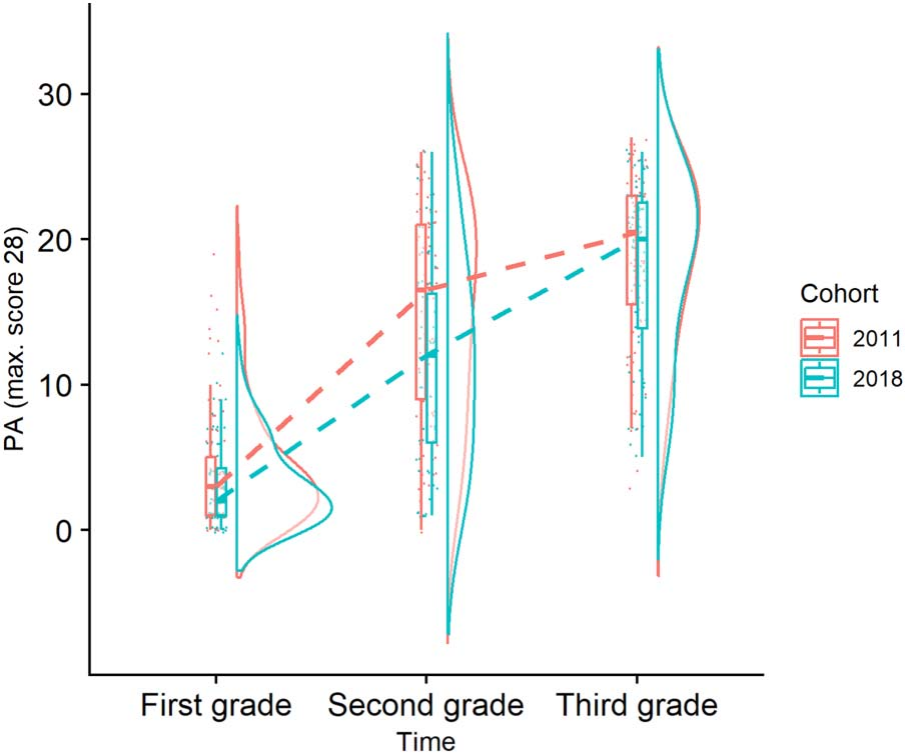
Growth in PA from first to second to third grade as a function of cohort (dashed line connects median values over time). For the 2018 cohort the first grade time point is before the school closures, the second grade time point closely after the school closures, and the third grade time point more than one year after the school closures.

First, we examined the growth in PA from first to second to third grade (Figure 1), where a decreased PA growth related to COVID-19 school closures would be reflected in a significant interaction between time and cohort. As can be seen from Table 2, we found a significant positive effect of time (large effect sizes) and cohort as well as a cohort-by-time interaction effect. In line with the pattern seen in Figure 1, post hoc investigation of this interaction effect showed that a significant difference between cohorts was found at the Grade 2 time point (directly after the school closures; *F* (1, 153) = 8.028, *p* = 0.005), but not at Grade 1 (prior to school closures; *F* (1, 157) = 3.307, *p* = 0.071) and at the Grade 3 (more than one year after school closures; *F* (1, 151) = 0.357, *p* = 0.551) time point. The Shapiro-Wilk test of the model residuals was not significant (*W* = 0.99, *p* = 0.188), indicating that the normality assumption was met. Adding the sex, family risk, and parental education covariates confirmed the main effect of time and cohort, but the cohort-by-time interaction no longer reached significance (*p* = 0.076). We also found an additional positive effect of parental education and family risk.

**Table 2.** Standardized estimates and *F* and *p* values for PA analyses.

Predictor	*F*	*p*
Time	466.59	<0.001*
Cohort	5.55	0.019*
Time:cohort	4.02	0.019*

* *p* < 0.05.

**Figure 2.**
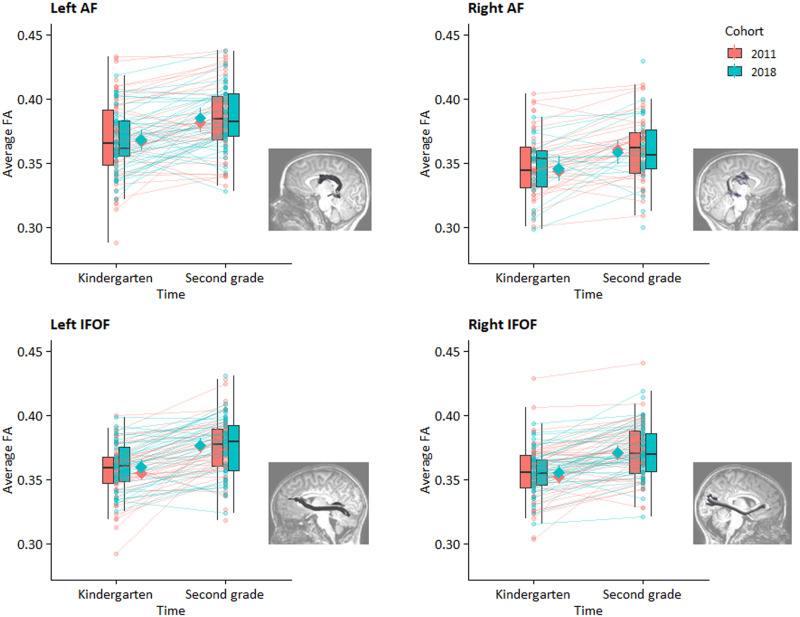
Tract average harmonized FA values as a function of time and grouped by cohort. The solid diamonds represent model-predicted FA values with 95% confidence intervals. Tracts are visualized next to each plot. Figures for non-harmonized FA values can be found in Supplementary Material.

Next, we looked at the longitudinal development of white matter structure in AF and IFOF from the end of kindergarten to the end of second grade and how this was influenced by the school closures. For the 2018 cohort school closures happened in Grade 1, hence we examine here the longer term effects (i.e., one year later) of COVID school closures. Head motion was included as covariate in these analyses. A similar pattern of effects was found in each of the four tracts (see Table 3). As can be seen from the visualization of the harmonized FA values in Figure 2, the mixed effects models showed a significant positive main effect of time (i.e., FA increased from kindergarten to second grade) and no significant main effect of cohort. There was no cohort-by-time interaction effect, indicating that the development of FA over time was not different between the two cohorts. These effects were found in the presence of a significant negative effect of motion on FA of the right AF and bilateral IFOF. The normality test was not significant for the left AF (*W* = 0.99, *p* = 0.306), the right AF (*W* = 0.99, *p* = 0.548), and the right IFOF (*W* = 0.99, *p* = 0.235). However, since it was significant for the left IFOF (*W* = 0.98, *p* = 0.029), we performed a robust mixed model analysis which revealed a similar pattern of results (*p*_time_ = <0.001, *p*_cohort_ = 0.461, *p*_interaction_ = 0.548, *p*_motion_ = <0.001). Adding the covariates sex, family risk, parental education and age at the end of kindergarten did not change the pattern of results. We did find an additional significant effect of sex in the left AF (higher FA in boys compared to girls).

**Table 3.** Standardized estimates, *F* and *p* values for average fractional anisotropy analyses of the left arcuate fasciculus (AF), right AF, left inferior fronto-occipital fasciculus (IFOF) and right IFOF.

Tract	Predictor	*β*	*F*	*p*
LAF	Time	0.25	37.96	<0.001*
	Cohort	0.03	0.28	0.596
	Time:cohort	0.03	0.18	0.676
	Motion	−0.09	2.32	0.129
RAF	Time	0.29	24.72	<0.001*
	Cohort	0.01	0.06	0.812
	Time:cohort	−0.05	0.33	0.571
	Motion	−0.21	8.88	0.004*
LIFOF	Time	0.40	84.61	<0.001*
	Cohort	0.08	0.54	0.462
	Time:cohort	−0.04	0.36	0.549
	Motion	−0.28	25.82	<0.001*
RIFOF	Time	0.36	69.65	<0.001*
	Cohort	0.03	0.10	0.747
	Time:cohort	−0.01	0.03	0.853
	Motion	−0.20	12.21	<0.001*

* *p* < 0.05.

In the Supporting Information, results of the node-based (Tract Profile) analyses can be found (Supplementary Material 2). In short, these analyses did not reveal a cohort-by-time interaction effect in any cluster of multiple adjacent nodes either, except for a single anterior node in the left IFOF. Next, supplementary analyses for ILF (see Supplementary Material 3) revealed the same pattern of results as for AF and IFOF, namely no cohort-by-time interaction effect. In addition, when imputation techniques are used to deal with missing data (largely due to failed tract reconstruction via AFQ), we again found no evidence for a cohort-by-time interaction (see Supplementary Material 6). Finally, to ensure that the absence of a cohort-by-time interaction effects on white matter is not due to differences between cohorts in pre-COVID cognitive skills, we performed supplementary analyses, where we re-ran the white matter mixed effects models in a subset of participants that were matched on pre-reading cognitive abilities, including only children with a cognitive risk for dyslexia (*n* = 30 for the 2011 cohort and *n* = 69 for the 2018 cohort). The pattern of results was identical in this subset of participants compared to the total sample (see Supplementary Material 4), confirming no cohort-by-time interaction and thus no effect of school closures.

## DISCUSSION

In the current longitudinal study, we investigated differences in the growth of PA and white matter connectivity in reading-related tracts (bilateral AF and bilateral IFOF) between a cohort of children who experienced COVID-19 school closures during first grade (the 2018 cohort) and a cohort of children who did not (the 2011 cohort). We found that PA and white matter connectivity in the four tracts increased over time. In addition, although there was a short-term impact of the school closures on the development of PA from Grade 1 to Grade 2, we did not find evidence for a long-term effect (i.e., more than 1 year after the school closures) on the growth of PA and white matter connectivity of reading-related tracts.

### Reading-Related Skills

PA was measured before (beginning Grade 1), directly after (beginning Grade 2), and more than 1 year after (beginning Grade 3) the school closures in the 2018 cohort, and the growth of PA was compared to the growth of PA in the 2011 cohort who did not experience school closures. We found clear evidence for an improvement of PA over time but also a significant time-by-cohort interaction effect, meaning that PA development was different for each cohort. More specifically, while cohorts did not differ significantly prior to (Grade 1) and more than one year after (Grade 3) the school closure, cohorts did differ significantly shortly after the school closures (Grade 2). This implies that there was a short-term effect of the school closure, but children of the 2018 cohort caught up with their peers of the 2011 cohort by the beginning of Grade 3. We expected an effect of school closures on PA development based on (1) the bidirectional relationship between PA and reading (Clayton et al., 2020; Peterson et al., 2018), which depends partially on schooling (Peng & Kievit, 2020); (2) the growing body of evidence for negative school closure effects on reading (Betthäuser et al., 2023; Hammerstein et al., 2021); and (3) at least one study reporting a negative impact on PA (Coskun & Kara, 2022). However, evidence is not univocal as other studies did not always find a negative effect on reading (Depping et al., 2021; Förster et al., 2023; Gore et al., 2021; Kuhfeld, Tarasawa, et al., 2020) or PA (Sun et al., 2023). Our study seems to indicate that the time gap between the school closures and the PA measurements is determinant for finding an effect, as we observe short-term (i.e., few months after the school closure) but no long-term (i.e., more than one year after the school closure) effects on reading-related skills.

Possible explanations for conflicting findings across studies include length of school closures (Donnelly & Patrinos, 2022), the quantity and quality of at-home schooling/support (Hammerstein et al., 2021), country income level, and parental education level (Betthäuser et al., 2023). In Flanders, school closures were relatively short (from March 2020 until May 2020), and although schools generally implemented at least some form of online teaching/learning activities, it is very likely that the quantity and content varied substantially across schools. To investigate individual differences within cohort 2018, we provide exploratory analyses in Supplementary Material 5 where questionnaire-based variables on the amount of shared reading and parental support during school closures as well as the duration of the school closures were related to growth in PA. This showed that the amount of parental support with homework during the school closure was related to the growth in PA from first to second grade (*r* = 0.27, *p* = 0.028) and from first to third grade (*r* = 0.27, *p* = 0.029), suggesting that parents might have played a role in improving reading-related skills of their child during school closures.

### White Matter Development in the Reading Network

Reading development has been associated with structural and functional changes in a left hemispheric reading network (Chyl et al., 2021) at a time when the brain is considered to be very sensitive to external influences (Nelson & Gabard-Durnam, 2020). Therefore, we also looked at two crucial white matter tracts of the reading network, the AF and IFOF. As expected, we found that, as an index of white matter organization, FA increased over the two years in both cohorts for IFOF and AF (though note that for AF the time effect was not confirmed when using imputation techniques for missing data; see Supplementary Material 6). This increase in FA over time is compatible with maturation of these specific white matter tracts during early childhood (Dimond et al., 2020; Economou et al., 2022; Lebel & Beaulieu, 2011; Reynolds et al., 2019; Vanderauwera et al., 2017) and was found despite significant head motion effects in most tracts. Thus, in light of the methodological difficulties associated with childhood neuroimaging (Turesky et al., 2021), our findings confirm the importance of including head motion when analyzing brain measures (Baum et al., 2018; Yendiki et al., 2014). In addition, the node-based analyses in the Supplementary Information show that developmental effects are found at particular locations of the white matter tract. Although current cross-sectional and developmental studies have mainly investigated developmental effects for the average FA per tract (DiPiero et al., 2023; Lebel et al., 2019), future studies could also examine localized age effects.

Apart from the main effect of time, we found no cohort-by-time interaction effect for AF and IFOF, meaning that the FA increase was similar across both cohorts. Additional analyses (see Supplementary Material 3) for ILF, which has also been considered part of the reading network (Yeatman, Dougherty, Ben-Shachar, & Wandell, 2012), confirmed this pattern of results. In other words, we did not find evidence that the school closures impacted FA development over time in any of the tracts. Note, however, that our second MRI measure took place one year after the school closures, hence we were only able to examine long-term effects and not what the impact on the brain was directly after the school closures. Similar to our behavioral PA measure, for which we observed short-term but no long-term school closure effects, there might have been an effect directly after the school closures that we were unable to measure. Therefore, we cannot conclude that the school closures have not affected the brain at some point, but it seems that at least at the long term (one year after the school closures) the decrease of direct, in-person reading instruction due to the school closures did not negatively impact the development of structural connectivity. In a similar vein, in the 2018 cohort we also did not find a relation between the degree of white matter growth and the amount of parental support or the duration of the school closures (see Supplementary Material 5).

Although we find no evidence for an effect of COVID-19 school closures on white matter plasticity in the reading network, we identify at least two interesting avenues for future research. First, the COVID-19 pandemic could be considered an adverse event (McManus & Ball, 2020) and other early adverse experiences such as stress, abuse, or neglect have been related to altered neuroplasticity across the brain (Bick & Nelson, 2016; Nelson & Gabard-Durnam, 2020; Tooley et al., 2021). Since school closures and other measures to contain the pandemic are very likely accompanied by increased feelings of stress and social isolation, it could be that effects are represented in areas of the brain related to psychosocial well-being rather than academic development (Bzdok & Dunbar, 2022). To the best of our knowledge, two studies described an effect of the COVID-19 pandemic on adolescents’ gray matter, showing an accelerated maturation specifically in the bilateral hippocampus and amygdala (Gotlib et al., 2022) and in the hippocampus and prefrontal cortex (van Drunen et al., 2023). These regions have been related to psychosocial aspects and are especially sensitive to childhood stress (Cameron et al., 2017; Hart & Rubia, 2012; Tottenham & Sheridan, 2010). It is of interest for future studies to investigate the possible impact on other brain regions or networks further. Second, reading instruction changes due to school closures reflect a change in the educational environment, much like reading instruction increases due to interventions. In this regard, whether or not an influence of the changed environment on the brain can be observed might depend on the selected brain measure. While Economou et al. (2022) did not observe an impact of a preventive reading intervention in kindergartners on FA, they did find intervention effects on a more specific biologically informed measure of white matter myelination (Economou et al., 2023). Huber et al. (2018, 2021) reported the opposite pattern in older children, with clear effects of reading intervention on white matter mean diffusivity (MD) and to a lesser extent on FA, but not on a myelination measure. In the current study we did not include MD as outcome measure, hence we cannot test whether MD is more sensitive than FA to environmental-related changes (Huber et al., 2018). However, a very recent study in young children using a school cut-off design showed no evidence for an increased sensitivity of MD (relative to FA) to environmental-related white matter changes (Vandecruys et al., 2024). Concerning functional MRI measures, the recent meta-analysis of Perdue et al. (2022) could not find consistent evidence of functional neuroplasticity in children who received increased reading instruction (i.e., interventions beyond the typical school curriculum). In sum, it remains unclear which brain measure is most sensitive to detect environmental effects on the brain.

Finally, although diffusion MRI data of both cohorts were acquired using the same scanner type and imaging sequences, the MRI scanner itself was different for each cohort, hence we harmonized the data using NeuroComBat (Fortin et al., 2017). In the Supplementary Material we provide the results of the analyses on the FA-values without harmonization, which confirm the absence of a time-by-cohort interaction effect but now also show a main effect of cohort. Given that both cohorts are well matched (see also additional analyses in Supplementary Material 4) and that it is unlikely that FA in young children has evolved between 2011 and 2018, we assume that the FA difference between cohorts is due to scanner differences. Hence, we applied harmonization, resulting in the cohort effect being no longer present. The supplementary node-based analyses suggested that the cohort difference was localized in several clusters along the entire length of the four tracts, in agreement with the study of Mirzaalian et al. (2016), who found that scanner differences could be region specific. Unfortunately, inter- and intra-scanner reliability data were not available, making it challenging to determine if the statistically significant FA difference between the cohorts on the non-harmonized data is also practically significant. Coefficients of variation for FA between and within scanners reported in other studies go from 2.5% up to approximately 8% (Deprez et al., 2018; Prohl et al., 2019) but do not allow a direct comparison to the cohort differences that we found. The FA difference between cohorts suggest that harmonization techniques (Cetin-Karayumak et al., 2020; Fortin et al., 2017; Huynh et al., 2019) might be needed not only for multisite datasets with *different* acquisition parameters and scanners of *different* vendors, but also when the scanner is upgraded or a new one of the same type is installed.

### Limitations of the Current Study

A few limitations of the current study should be considered. First, the study sample was originally recruited based on family risk and cognitive risk criteria. This led to a sample of children overrepresented for dyslexia risk, of which only 36 out of 162 participants did not have any risk according to the predefined categorical inclusion criteria. A study in Germany reported that replacement activities for the reduced in-person teaching varied depending on academic performance prior to the school closures with high-achieving students engaging more in activities advantageous to development (i.e., reading, creative activities, and physical exercise) compared to low-achieving students (Grewenig et al., 2021). In a similar vein, Schult et al. (2022) found varying effects of school closures depending on academic performance prior to the pandemic. Therefore, it could be expected to find a negative school closure impact in only a subsample of children who had low cognitive performance prior to the school closures. Therefore, we re-ran our analyses on a subset of participants who were matched for cognitive performance on reading-related tasks prior to the school closures (i.e., only including children with a cognitive risk for dyslexia), which again revealed no cohort-by-time interaction effect and thus no evidence for a school closure effect on white matter connectivity. In other words, even in children who are arguably the most vulnerable (low cognitive performance prior to school closures), we did not find evidence for a negative impact of the COVID-19 school closures. The analyses in the cognitive risk subsample can be found in Supplementary Material 4. Second, given the specific characteristics of our study sample (Dutch-speaking children from a high-income country with comparable educational quality across schools and a relatively short period of full-time school closures), we cannot directly generalize these findings to pupils speaking other languages in other schooling systems who experienced longer school closures. Future research should continue to investigate the impact of COVID-19 school closures, whether positive, negative, or absent, to find out which findings replicate robustly in independent samples (Thomason, 2022).

Finally, for the reconstruction of the white matter tracts, we used deterministic and automated fiber tracking via AFQ, which did not succeed in reconstructing all tracts for all children. Hence, this resulted in missing data up to 20% for left AF and bilateral IFOF and even up to 50% for right AF. These percentages, and the higher percentage of missing data in right AF, are in line with previous developmental MRI studies using AFQ (Banfi et al., 2019; Economou et al., 2022; Langer et al., 2017; Van der Auwera et al., 2021), but urge for caution in interpreting the results. Given that these failed reconstructions have been attributed to limitations of deterministic and tensor-based algorithms in regions of crossing fibers rather than the anatomical absence of the tract from the brain (Duan et al., 2015; Yeatman et al., 2011), future studies should acquire diffusion data that allow better for constrained spherical deconvolution and automated (probabilistic) fibertacking, such as TractSeg (Wasserthal et al., 2018). Yet note that our additional analyses—where imputation techniques were used for the missing data (Supplementary Material 6)—show again no significant time-by-cohort interaction, hence our main conclusion that there is no evidence for a COVID-19 school closures effect on white matter plasticity in the reading network is confirmed.

### Conclusion

In summary, the current longitudinal study confirmed a different PA development related to COVID-19 school closures during a pivotal time of reading development (first year of formal reading instruction), but the effect was present only directly after school closures and no longer present one year later. For white matter connectivity within the reading network, we did not find a different white matter development in the cohort that experienced school closures, although it is important to note that the follow-up brain scan took place not directly after the school closures but one year later. Hence, we cannot exclude that short-term effects of school closures might have been present, but at the long term (end of Grade 2, which is one year after the school closures in Grade 1) no COVID-19 school closures effect on the reading network was observed.

## ACKNOWLEDGMENTS

We thank all the children and parents for their cooperation in our study. We would like to thank the researchers involved in longitudinal data collection of the first cohort: Sophie Dandache, Astrid De Vos, Jolijn Vanderauwera, and Sophie Vanvooren. We are also grateful to all students who assisted in data collection.

## FUNDING INFORMATION

Maaike Vandermosten, Fonds Wetenschappelijk Onderzoek (https://dx.doi.org/10.13039/501100003130), Award ID: G077018N.

## AUTHOR CONTRIBUTIONS

**Lauren Blockmans**: Conceptualization; Data curation; Formal analysis; Investigation; Methodology; Software; Visualization; Writing – original draft; Writing – review & editing. **Fumiko Hoeft**: Conceptualization; Writing – review & editing. **Jan Wouters**: Conceptualization; Funding acquisition; Resources; Supervision; Writing – review & editing. **Pol Ghesquière**: Conceptualization; Funding acquisition; Resources; Supervision; Writing – review & editing. **Maaike Vandermosten**: Conceptualization; Funding acquisition; Methodology; Resources; Supervision; Writing – review & editing.

## DATA AND CODE AVAILABILITY STATEMENT

The analyses script and anonymized data sheet are available at the Open Science Framework (https://osf.io/cekp6/).

## Supplementary Material



## TECHNICAL TERMS

COVID-19 lockdown: Encompasses stay-at-home orders, quarantines, and societal restrictions, as well as school closures.
Fractional anisotropy: Measures how much the water diffusion is directionally restricted; indirectly, it can provide information on the organization and density of white matter fibers in the brain.
