# Supplementary material for: Impact of COVID-19 School Closures on White Matter Plasticity in the Reading Network
Source: Neurobiol Lang (Camb). 2025 Jan 10;6:nol_a_00158. doi: 10.1162/nol_a_00158 (PMC11740157; doi:10.1162/nol_a_00158)
Supplement: Supplementary file 1 [file nol-6-nol_a_00158-s001.pdf]

Supplemental information

Supplementary Material 1: non-harmonized FA values

Given that data of each cohort were acquired on the same type of scanner (Philips Achieva) and with the same acquisition sequence, it was less clear whether harmonization techniques, which are normally applied to multi-site studies (where different scanner types and acquisition sequences are used), were also applicable. We therefore provide here in Supplementary Materials additional analyses on the 'raw' non-harmonized FA data. Mixed effects models showed a significant positive main effect of time (i.e., FA increased from kindergarten to second grade), and in contrast to the harmonized data in the main text, a significant main effect of cohort, in which the 2011 cohort had overall higher FA values. There was again no cohort-by-time interaction effect, confirming that the development of FA over time was not different between the two cohorts. These effects were found in the presence of a significant negative effect of motion on FA of the right AF and bilateral IFOF. Adding the covariates sex, parental education, family risk and age at the end of kindergarten did not change the pattern of results, but we did find an additional significant effect of sex in the left AF (higher FA in boys compared to girls).

Tract	Predictor	β	F	p
LAF	Time	0.23	37.64	<.001*
	Cohort	-0.42	29.45	<.001*
	Time:cohort	0.01	0.03	.140
	Motion	-0.08	2.20	.873
RAF	Time	0.23	25.07	<001*
	Cohort	-0.45	25.25	<001*
	Time:cohort	-0.00005	0.00	.999
	Motion	-0.19	9.98	.002*

Tract	Predictor	β	F	p
LIFOF	Time	0.27	85.90	<.001*
	Cohort	-0.51	38.48	<001*
	Time:cohort	0.13	3.94	.051
	Motion	-0.25	25.70	<.001*
RIFOF	Time	0.29	69.98	<001*
	Cohort	-0.43	32.52	<001*
	Time:cohort	0.05	0.61	.435
	Motion	-0.19	12.66	<.001*

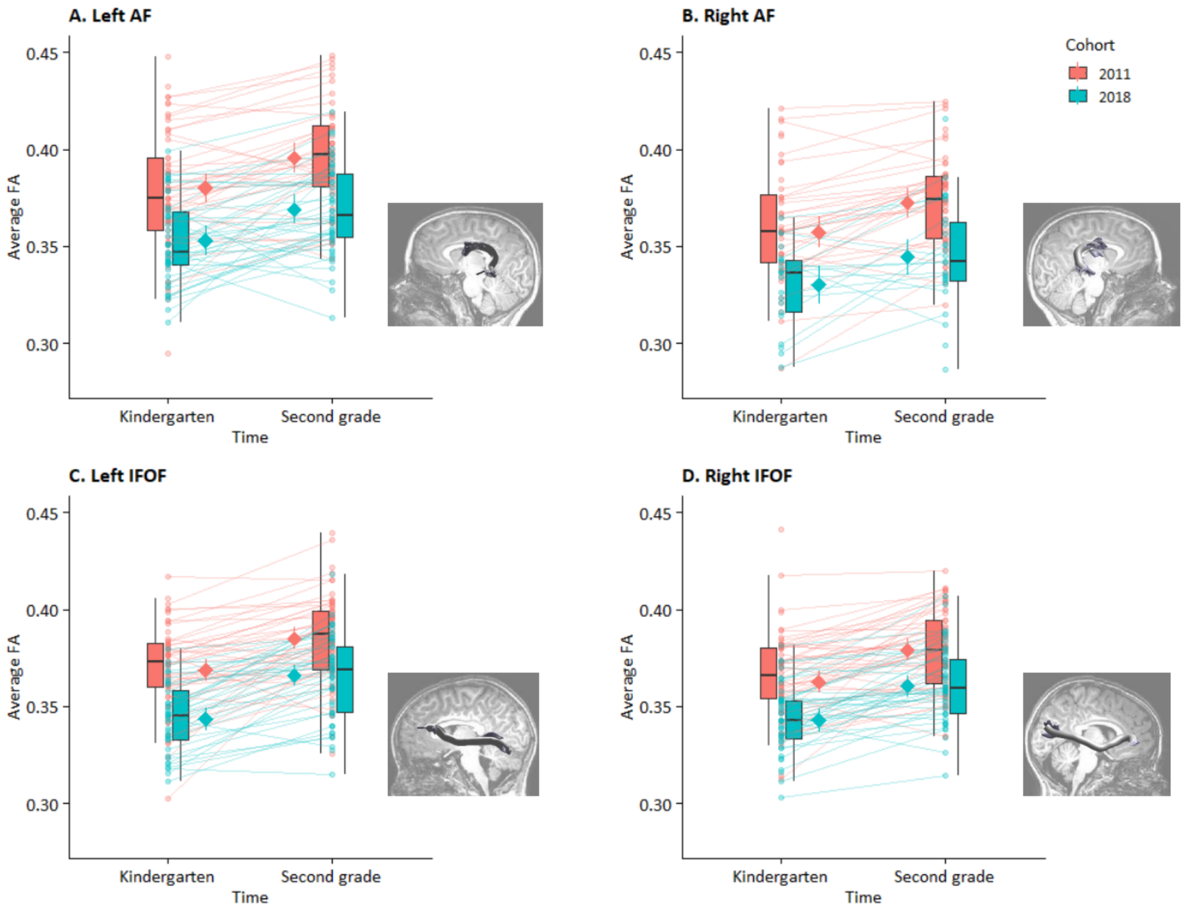

Supplementary Material 2: Tract Profile analyses

Node-based (Tract Profile) analyses were performed as an extension of the tract average analyses. As described under 2.3, the AFQ pipeline includes a feature to sample each tract into a predefined number of locations (nodes) that are spaced equally along the tract. By default, FA is calculated at 100 nodes. In the current study, AFQ could not calculate FA at the first and last node of the tracts, therefore only nodes 2 until 99 were retained. To analyze the effects of time, cohort, head motion and family risk at each node, a suitable correction for multiple comparisons was required. Since adjacent nodes are highly correlated, and thus not independent, a Bonferroni correction would be too strict (Yeatman, Dougherty, Myall, et al., 2012). Instead, we used a non-parametric permutation-based correction (Nichols & Holmes, 2001) which was recommended in the AFQ pipeline (function 'AFQ_MultiCompCorrection') and applied in several recent papers (Economou et al., 2022; Farah et al., 2020; Jossinger et al., 2021; Wasserthal et al., 2021). For this correction method, which was applied to each tract separately, the subjects were first randomly assigned to the two cohorts. Then, test statistics and *p*-values were calculated for each node along the tract and the lowest *p*-value was retained. This was repeated 1000 times (permutations), after which the family-wise error (FWE) corrected cluster size threshold at $\alpha = .05$ was calculated. Additionally, a FWE-corrected alpha value was calculated at the 95th percentile (i.e., 50th largest *p*-value) of the statistics distribution across the 1000 permutations. Effects that occurred at nodes with a *p*-value below the the FWE-corrected alpha, or effects that occurred in at least as many adjacent nodes as the FWE-corrected cluster size, were considered significant effects.

FWE-corrected alpha thresholds for the effects of time, cohort, cohort-by-time interaction, motion and family risk in the different tracts were maximum $p = .0000009$, $p = .003$, $p = .002$, $p = .005$ and $p = .037$ respectively, while the FWE-corrected cluster sizes ranged between 34-61, 10-13, 8-10, 12-29 and 1-6, nodes respectively.

The node-based results suggested that the main effects of cohort (Supplementary Figure 2A and 2B) were located in several clusters along the entire length of the four tracts, but revealed no specific portions of the tracts where the effects of time (Supplementary Figure 3A and 3B) were located, except for one large cluster in the right IFOF. There were also no clusters of nodes with a significant cohort-by-time interaction effect (Supplementary Figure 4A and 4B), except for one single anterior node (node 9) in the left IFOF, or a head motion effect (Supplementary Figure 5A and 5B). A family risk effect (Supplementary Figure 6A and 6B) was present in a few relatively small clusters of the left AF and the right IFOF. In the left AF (Supplementary Figure 7A), we found a negative effect in nodes 23-26 indicating that a higher parental ARHQ score (i.e., more self-reported difficulties in parents' reading history) was associated with lower FA in that cluster. In the right IFOF (Supplementary Figure 7B), we found a negative family risk effect in nodes 35-40 indicating that a higher parental ARHQ score was associated with lower FA in that cluster.

Supplementary Figure 2: *Uncorrected p-values (A) and Cohen's f effect sizes (B) for the effect of cohort.*

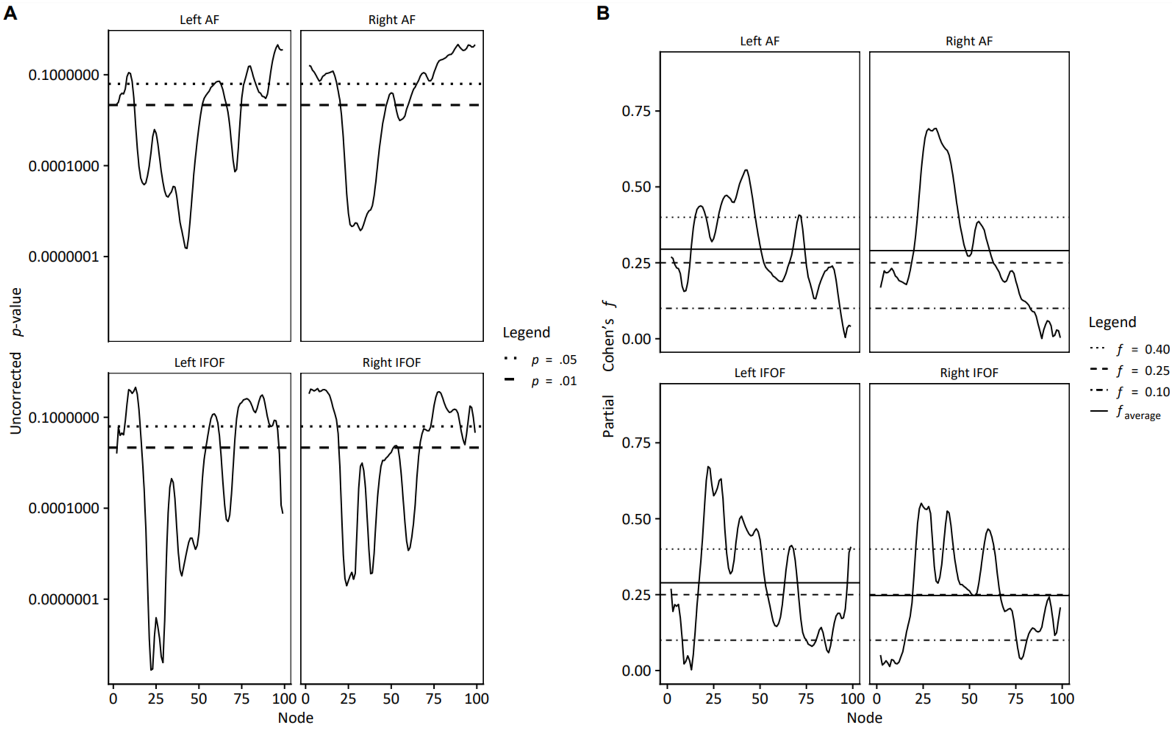

Supplementary Figure 3: *Uncorrected p-values (A) and Cohen's f effect sizes (B) for the effect of time.*

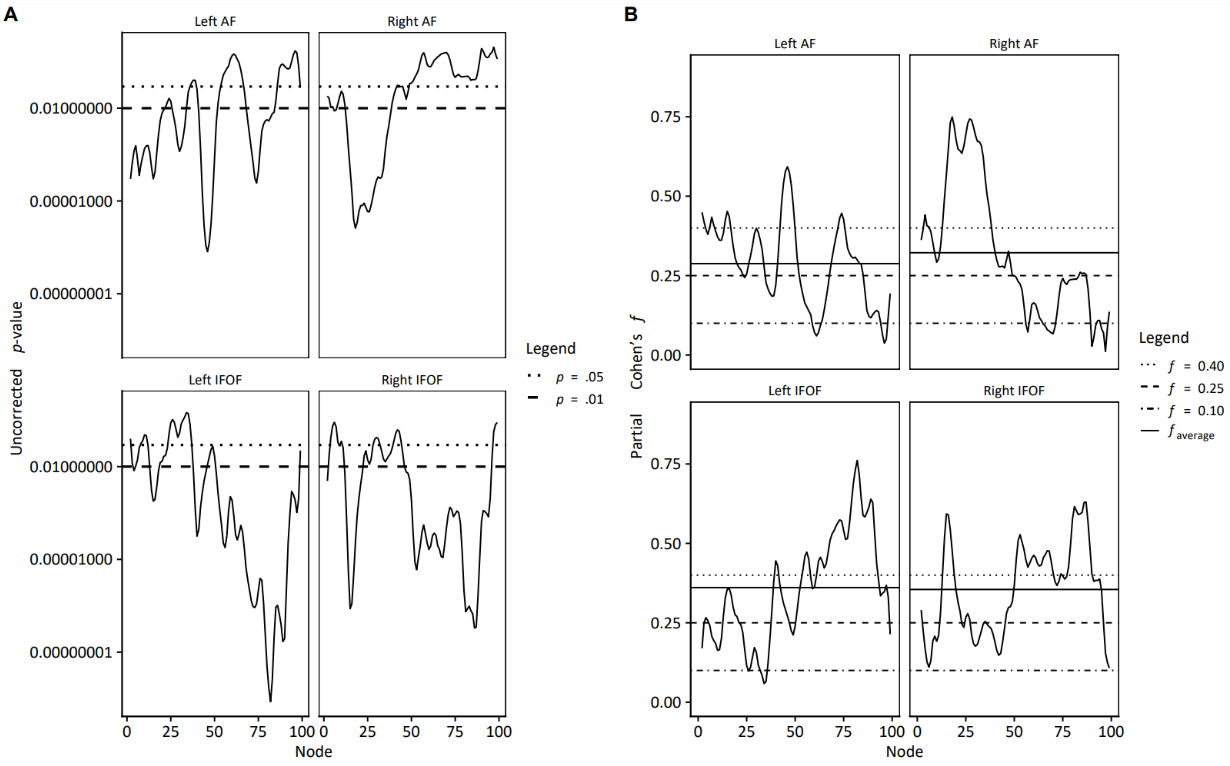

Supplementary Figure 4: *Uncorrected p-values (A) and Cohen's f effect sizes (B) for the effect of cohort-by-time.*

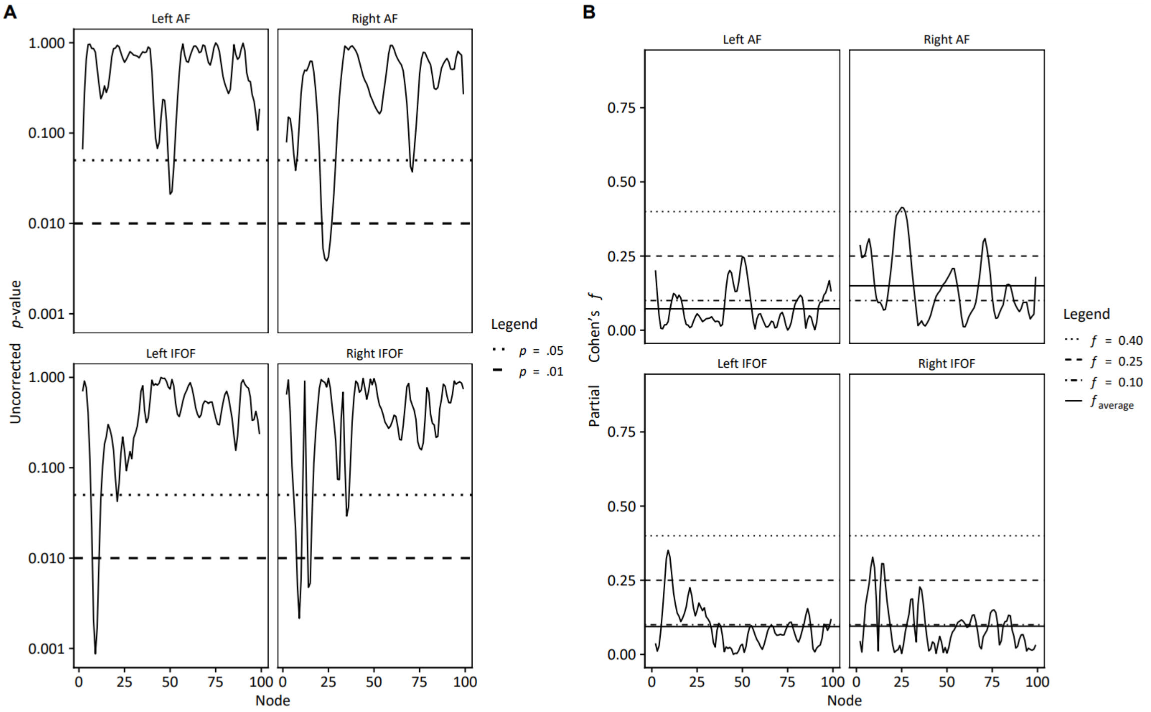

Supplementary Figure 5: *Uncorrected p-values (A) and Cohen's f effect sizes (B) for the effect of motion.*

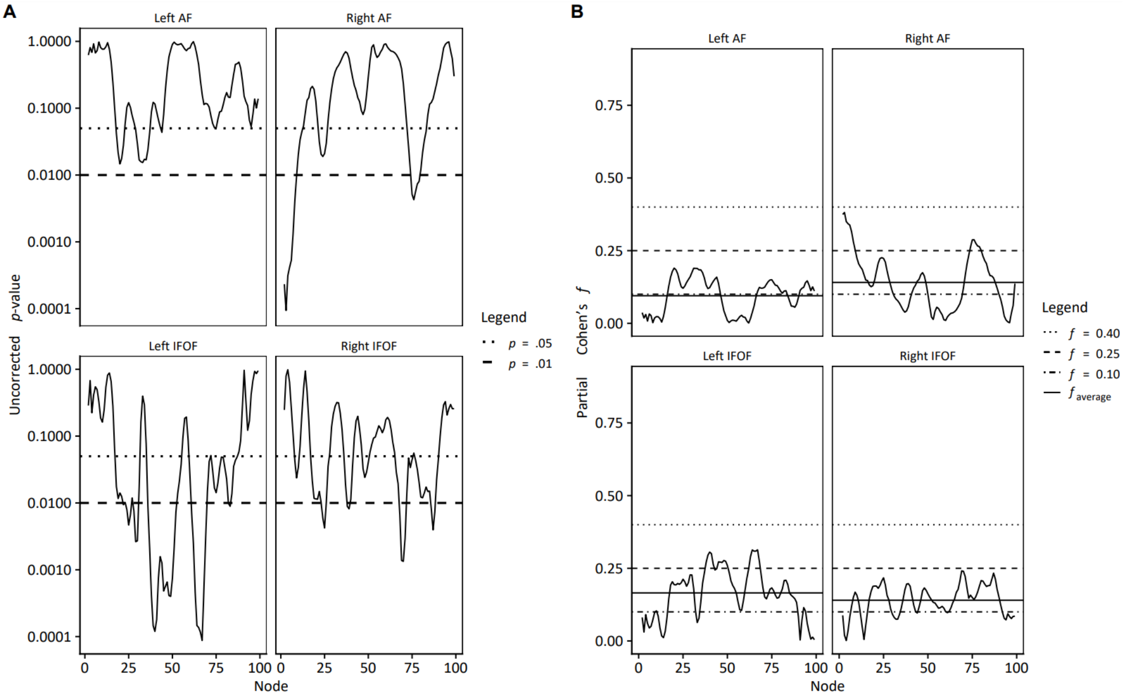

Supplementary Figure 6: *Uncorrected p-values (A) and Cohen's f effect sizes (B) for the effect of family risk.*

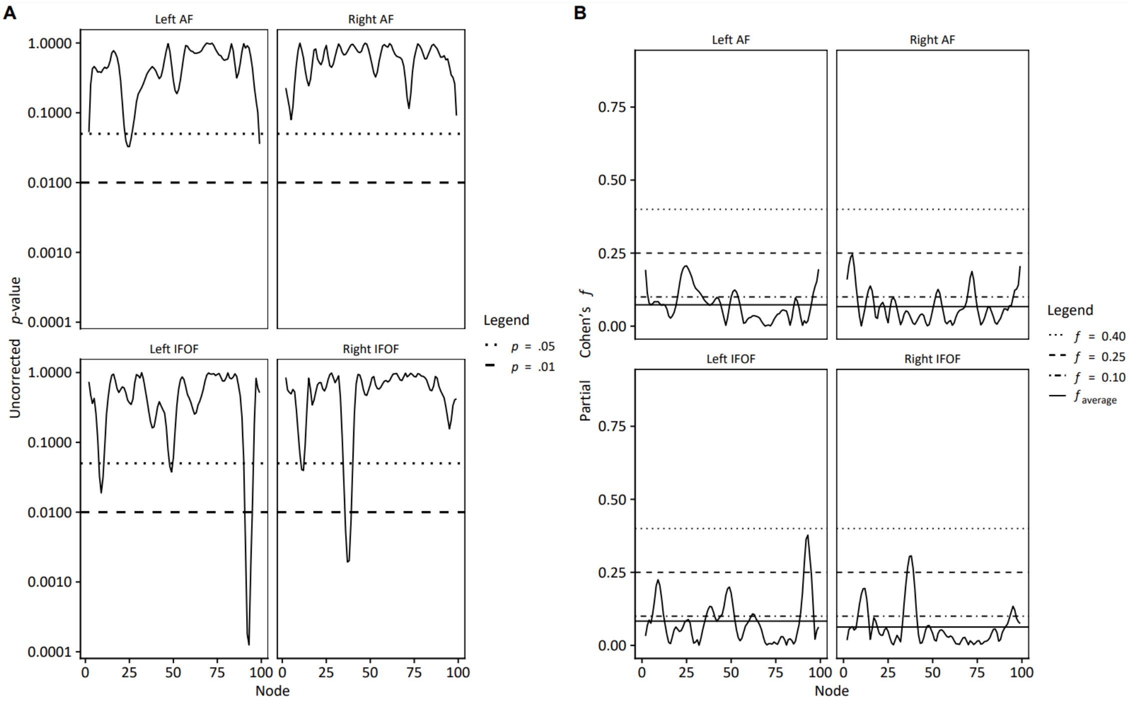

Supplementary Figure 7: *Family risk clusters.*

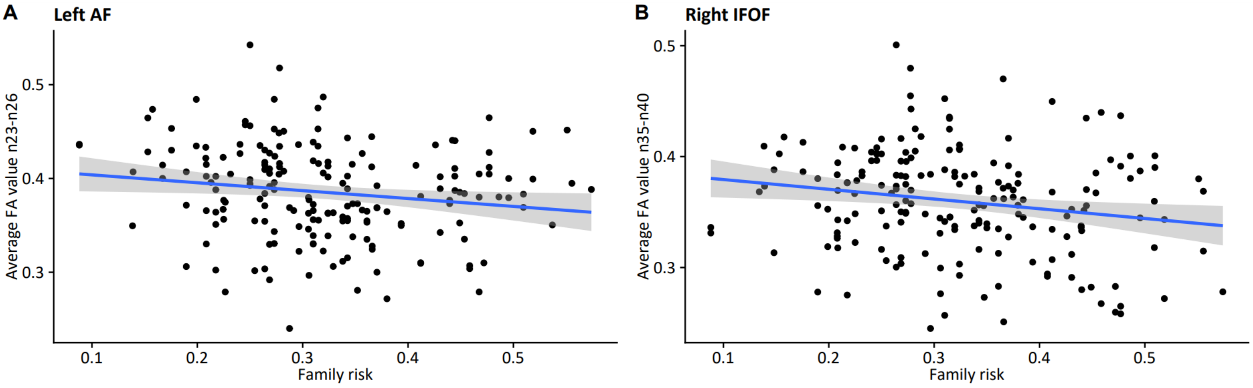

Supplementary Material 3: ILF

Although the focus of our study was specifically on bilateral AF and IFOF, in line with other studies (Yeatman et al., 2012?; Economou et al., 2022) we post-hoc performed additional AFQ analyses for ILF. As shown in the table below and the figure of ILF (harmonized data), the same pattern as in the other reading-related tracts was found, namely a main effect of time but no main effect of cohort and no cohort-by-time interaction effect.

Tract	Predictor	β	F	p
LILF	Time	0.37	62.16	<.001*
	Cohort	0.03	0.0009	.976
	Time:cohort	-0.06	0.64	.427
	Motion	-0.18	9.57	.002*
RILF	Time	0.32	42.43	<.001*
	Cohort	-0.0008	0.24	.626
	Time:cohort	-0.07	0.81	.371
	Motion	-0.19	10.14	.002*

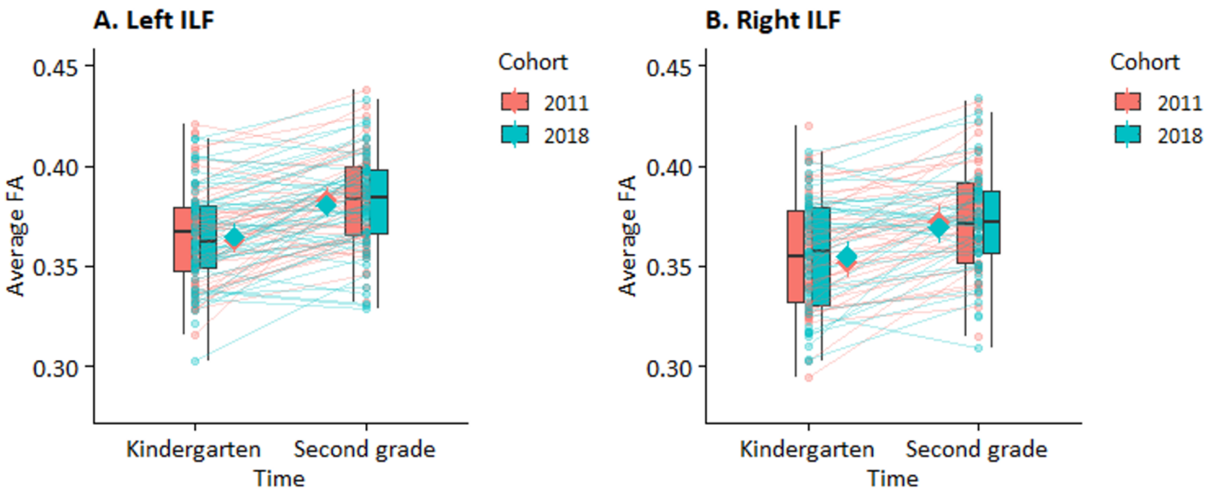

Supplementary Material 4: cognitive risk subsample

For these additional analyses, we included a subsample of the total cohort, selecting only children with a cognitive risk. The latter is defined as scoring below the 30th percentile on at least two out of three pre-reading tasks (PA, rapid automatized naming and letter knowledge. This resulted in 99 at risk children, of whom 30 belong to cohort 2011 and 69 to cohort 2018.

Supplementary Table 2: *Standardized estimates, F- and p-values for average FA of the left AF, right AF, left IFOF and right IFOF in the cognitive risk subsample.*

Tract	Predictor	β	F	p
LAF	Time	0.30	23.60	<.001*
	Cohort	-0.35	12.34	<.001*
	Time:cohort	-0.00	0.00	.977
	Motion	-0.14	4.00	.049*
	Family risk	-0.02	0.03	.855
RAF	Time	0.19	5.98	.021*
	Cohort	-0.36	5.33	.026*
	Time:cohort	0.07	0.15	.700
	Motion	-0.25	5.56	.022*
	Family risk	0.05	0.10	.750
LIFOF	Time	0.40	53.88	<.001*
	Cohort	-0.30	7.86	.007*
	Time:cohort	0.06	0.29	.591
	Motion	-0.26	13.98	<.001*
	Family risk	-0.04	0.16	.692
RIFOF	Time	0.39	40.25	<.001*
	Cohort	-0.25	7.69	.007*
	Time:cohort	0.00	0.00	.999

Tract	Predictor	β	F	p
	Motion	-0.30	17.22	<.001*
	Family risk	-0.02	0.07	.793

Supplementary Material 5: individual differences within the 2018 cohort

We administered a questionnaire in cohort 2018 to gain information on individual differences in the amount of support during the school closures and the duration of the school closures. This allows to examine relations with the amount of growth in PA and white matter. Note that our sample size for these analyses is rather small, given that the analyses are restricted to cohort 2018 and, for white matter, to the ones that have both MRI measures ((LAF n = 38, RAF n = 16, LIFOF n = 34, RIFOF n = 36)). Results show only a significant correlation between the amount of parental support with homework during the school closures and the growth in PA.

For PA change:

- Shared reading (in minutes) during school closures does not correlate with growth in PA from first to second grade (r = -0.07, p = .567) and from first to third grade (r = -0.03, p = .803).
- Amount of parental support with homework during school closures correlates with growth in PA from first to second grade (r = 0.27, p = .028) and from first to third grade (r = 0.27, p = .029).
- Re-opening of schools does not correlate with growth in PA from first to second grade (r = -0.01, p = .919) and from first to third grade (r = 0.09, p = .439).

For white matter change:

- Shared reading (in minutes) during school closures does not correlate with growth in FA of LAF (r = 0.07, p = .697), RAF (r = 0.34, p = .205), LIFOF (r = -0.30, p = .089) or RIFOF (r = 0.01, p = .935) from T1 to T2.
- Amount of parental support with homework during school closures does not correlate with growth in FA of LAF (r = 0.15, p = .383), RAF (r = 0.22, p = .420), LIFOF (r = 0.09, p = .594) or RIFOF (r = -0.21, p = .208) from T1 to T2.
- Re-opening of schools does not correlate with growth in FA of LAF (r = -0.03, p = .840), RAF (r = -0.25, p = .347), LIFOF (r = 0.21, p = .226) or RIFOF (r = -0.05, p = .779) from T1 to T2.

Supplementary Material 5: imputation techniques for missing data in white matter tracts

We used automated fiber quantification (AFQ) to characterize the selected white matter tracts, but this resulted in a substantial amount of failed tract reconstructions. Using AFQ, the proportion of missing data across both time points was below 20% for left AF and bilateral IFOF and up to 50% for right AF (for more details see Methods). To test whether the observed pattern of results, in particular our main finding of a non-significant time-by-cohort interaction, was not biased by our missing data, we conducted multiple-imputation, using BLIMP software in R (: https://www.appliedmissingdata.com/blimp). The obtained potential scale reduction factors (PSR) were all below 1.05, indicating good convergence of the models for each white matter tract.. As indicated in the tables below, although the main effect of time was no longer significant relative to the results in the main text (using no imputation), the most crucial effect, namely the time-by-cohort interaction, was again not significant for bilateral AF and IFOF (*p* > .447), hence confirming that our study provides no evidence for a COVID19 school closure effect on the development of the white matter reading network.

**Tract**	**Predictor**	**Estimate**	**Std. error**	**t-value**	**df**	**p**
LAF	Time	0.30	0.29	1.02	193.1	.309
	Cohort	-0.08	0.31	-0.24	393.4	.810
	Time:cohort	0.14	0.19	0.76	186.9	.447
	Motion	-0.11	0.06	-1.68	244.5	.095
RAF	Time	0.42	0.28	1.51	151.2	.132
	Cohort	0.06	0.34	0.19	120.0	.851
	Time:cohort	0.03	0.19	0.15	116.6	.880
	Motion	-0.21	0.08	-2.75	61.6	.008
LIFOF	Time	0.71	0.24	2.94	489.3	.003*
	Cohort	0.18	0.29	0.62	311.2	.534
	Time:cohort	-0.03	0.16	-0.16	308.6	.872
	Motion	-0.31	0.06	-5.00	141.4	<.001*
RIFOF	Time	0.64	0.26	2.45	819.2	.015*
	Cohort	0.08	0.31	0.25	389.7	.806
	Time:cohort	0.02	0.17	0.09	551.0	.931
	Motion	-0.21	0.065	-3.31	177.7	.001

References

Brus, T., & Voeten, M. (1999). *Eén-minuut-test (EMT)*. Harcourt Test Publishers.

Economou, M., Van Herck, S., Vanden Bempt, F., Glatz, T., Wouters, J., Ghesquière, P., Vanderauwera, J., & Vandermosten, M. (2022). Investigating the Impact of Early

Literacy Training on White Matter Structure in Prereaders at Risk for Dyslexia. *Cerebral Cortex*, *00*, 1–14. https://doi.org/10.1093/cercor/bhab510

Farah, R., Tzafrir, H., & Horowitz-Kraus, T. (2020). Association between diffusivity measures and language and cognitive-control abilities from early toddler's age to childhood. *Brain Structure and Function*, *225*, 1103–1122. https://doi.org/10.1007/s00429-020-02062-1

Hammerstein, S., König, C., Dreisörner, T., & Frey, A. (2021). Effects of COVID-19-Related School Closures on Student Achievement-A Systematic Review. *Frontiers in Psychology*, *12*, 1–8. https://doi.org/10.3389/fpsyg.2021.746289

Jossinger, S., Kronfeld-Duenias, V., Zislis, A., Amir, O., & Ben-Shachar, M. (2021). Speech rate association with cerebellar white-matter diffusivity in adults with persistent developmental stuttering. *Brain Structure and Function*, *226*, 801–816. https://doi.org/10.1007/s00429-020-02210-7

Kloke, J. D., & McKean, J. W. (2012). Rfit: Rank-based estimation for linear models. *The R Journal*, *4*, 57–64.

Nichols, T., & Holmes, A. (2001). Nonparametric Permutation Tests for Functional Neuroimaging: A Primer with Examples. *Human Brain Function*, *15*, 1–25. https://doi.org/10.1016/B978-012264841-0/50048-2

Wasserthal, J., Maier-Hein, K. H., Neher, P. F., Wolf, R. C., Northoff, G., Waddington, J. L., Kubera, K. M., Fritze, S., Harneit, A., Geiger, L. S., Tost, H., & Hirjak, D. (2021). White matter microstructure alterations in cortico-striatal networks are associated with parkinsonism in schizophrenia spectrum disorders. *European Neuropsychopharmacology*, *50*, 64–74. https://doi.org/10.1016/j.euroneuro.2021.04.007

Yeatman, J. D., Dougherty, R. F., Myall, N. J., Wandell, B. A., & Feldman, H. M. (2012). Tract Profiles of White Matter Properties: Automating Fiber-Tract Quantification. *PLoS ONE*, *7*(11), e49790. https://doi.org/10.1371/journal.pone.0049790
